# Short-term health-related quality of life, physical function and psychological consequences of severe COVID-19

**DOI:** 10.1186/s13613-021-00881-x

**Published:** 2021-06-04

**Authors:** Luca Carenzo, Alessandro Protti, Francesca Dalla Corte, Romina Aceto, Giacomo Iapichino, Angelo Milani, Alessandro Santini, Chiara Chiurazzi, Michele Ferrari, Enrico Heffler, Claudio Angelini, Alessio Aghemo, Michele Ciccarelli, Arturo Chiti, Theodore J. Iwashyna, Margaret S. Herridge, Maurizio Cecconi, the Humanitas COVID-19 Task Force

**Affiliations:** 1 Department of Anesthesia and Intensive Care Medicine, Humanitas Clinical and Research Center-IRCCS, Via Manzoni 56, 20089 Rozzano, Italy; 2 Department of Biomedical Sciences, Humanitas University, Via Rita Levi Montalcini 4, 20090 Pieve Emanuele, Italy; 3 Personalized Medicine, Asthma and Allergy, Humanitas Clinical and Research Center-IRCCS, Via Manzoni 56, 20089 Rozzano, Italy; 4 Department of Renal Medicine, Humanitas Clinical and Research Center-IRCCS, Via Manzoni 56, 20089 Rozzano, Italy; 5 Department of Internal Medicine and Hepatology, Humanitas Clinical and Research Center-IRCCS, Via Manzoni 56, 20089 Rozzano, MI Italy; 6 Department of Respiratory Medicine, Humanitas Clinical and Research Center-IRCCS, Via Manzoni 56, 20089 Rozzano, Italy; 7 Humanitas Clinical and Research Center-IRCCS, Via Manzoni 56, 20089 Rozzano, Italy; 8 Division of Pulmonary and Critical Care Medicine, University of Michigan, Ann Arbor, USA; 9 Interdepartmental Division of Critical Care Medicine, Institute of Medical Science, Toronto General Research Institute, University of Toronto, Toronto, Canada

**Keywords:** COVID-19, Follow-up, 6-min walking test, Health Related Quality of Life, Post-Traumatic Stress Disorder

## Abstract

**Background:**

Survivors of severe COVID-19 are at risk of impaired health-related quality of life (HRQoL) and persistent physical and psychological disability after ICU and hospital discharge. The subsequent social burden is a major concern. We aimed to assess the short-term HRQoL, physical function and prevalence of post-traumatic stress symptoms of invasively mechanically ventilated COVID-19 patients treated in our ICU.

**Methods:**

Prospective, observational cohort study in a follow-up clinic. Patients completed a 6-min walking test (6MWT) to assess their cardio-pulmonary function around 2 months (early follow-up) from hospital discharge, the EQ-5D-5L questionnaire for quality of life assessment around 2 months and at 6 months from hospital discharge and an anonymous web-based Impact of Event Scale-Revised (IES-R) questionnaire for Post-Traumatic Stress symptoms at 2 months.

**Results:**

47 patients attended our follow-up program, mean age 59 ± 10 years, median pre-morbid Clinical Frailty Scale (CFS) 2 [2–3]. The median distance walked in 6 min was 470 [406–516] m, 83 [67–99]% of the predicted value. Overall 1 out 3 patients and 4/18 (22%) among those with a good functional baseline prior to COVID-19 (CFS of 1 or 2) had lower (84%) than predicted 6MWT. EQ-5D-5L quality of life VAS was 80 [70–90] out of 100 at early follow-up with a slight improvement to 85 [77.5–90] at 6 months. Mobility, self-care and usual activities improved between the two timepoints, while pain/discomfort and depression/anxiety did not improve or got worse. The IES-R total score was greater than the threshold for concern of 1.6 in 27/41(66%) respondents.

**Conclusions:**

Patients recovering from severe COVID-19 requiring invasive mechanical ventilation surviving hospital discharge present with early mild to moderate functional impairment, mildly reduced quality of life from hospital discharge with an overall improvement of mobility, self-care and the ability of performing usual activities, while a worsening of pain and depression/anxiety symptoms at 6 months and a large proportion of symptoms of post-traumatic distress soon after hospital discharge.

## Introduction

The novel coronavirus disease (COVID-19) has generated an extraordinary number of patients admitted to Intensive Care Units (ICU) for treatment of acute and severe respiratory failure [1, 2]. This and other critical illnesses associated with prolonged ICU stay are well known risk factors for reduction in health-related quality of life (HRQoL), physical function and psychological disability [3]. In a recent letter, patients with COVID-19, mostly treated outside of ICU, commonly suffered from fatigue, dyspnea or other symptoms, and reported worsened quality of life, after hospital discharge [4]. A recent case-series of severe COVID-19 cases (i.e., patients with hypoxemic respiratory failure requiring hospitalization) not requiring invasive mechanical ventilation concluded that outcomes might be better than expected, as patients were unlikely to develop long-term pulmonary impairment [5]. Several recent papers investigated short-term consequences of COVID-19 although in more heterogeneous populations: Goertz et al. [6] found a significant prevalence of fatigue and dyspnea at 3 months from symptoms onset but excluded ICU patients, similar symptoms were found in the work of Halpin et al. [7], the work of Belli et al. [8] is limited to inpatient rehabilitation symptoms, while the work of Arnold et al. [9] is limited by a 28-day follow-up. It is still unclear whether those treated in the ICU might be at higher risk of persisting pulmonary and extrapulmonary disability [10–12] and pertinent data are still lacking. This study aimed to describe the short-term HRQoL, physical function and prevalence of post-traumatic stress symptoms of invasively mechanically ventilated COVID-19 patients treated in our urban tertiary academic ICU (more details on our ICU and hospital response to COVID can be found in reference 2).

## Methods

In our institution, patients with laboratory-confirmed COVID-19 were invited to attend a follow-up program coordinated by the Infectious Disease service, approximately 2 months after hospital discharge. There, survivors of invasive mechanical ventilation > 48 h underwent a 6-min walking test (6MWT) and completed the 5-level (Italian) version of the EQ-5D questionnaire (EQ-5D-5L) under the direct supervision of a study investigator. Patients who were missed at follow-up (did not show up/research team not available) were invited to complete the questionnaire by phone. Finally, patients completed an anonymous web-based Impact of Event Scale – Revised (IES-R) questionnaire to assess the degree of symptoms of Post-Traumatic Stress. All patients were then phone interviewed again at 6 months following hospital discharge for their quality of life using the EQ-5D-5L. The selection of tools was based on the recommendations from the Improve Long Term Outcome Research after Acute Respiratory Failure work [13, 14]. Our institutional Ethics Committee approved this study (protocol number 465/20). Written informed consent was obtained from all patients before ICU discharge. The study was performed in accordance with Strengthening the Reporting of Observational studies in Epidemiology (STROBE) guidelines [15].

Characteristics of the inpatient stay were abstracted from hospital electronic health records. The Clinical Frailty Scale (CFS) [16] at hospital admission was recorded as routine practice by the coordinating physician and ascertained from a surrogate decision maker/family member.

The 6MWT evaluates the functional exercise capacity [17, 18]. The test was run as per American Thoracic Society standards, on a flat straight 30-m outdoor paved surface within the hospital premises. Continuous pulse-oximetry was applied. Heart rate and blood pressure were measured before and after the test, while difficulty in breathing was graded with the Borg scale [17]. The predicted values for a healthy person of the same sex, age, weight, and height were calculated as in Enright and Sherril [19].

The EQ-5D-5L questionnaire assesses the degree of difficulty in key health-related domains. Patients report their perceived problems from “none” to “extreme” in five domains (mobility, self-care, usual activities, pain and discomfort, anxiety and depression) and also rate their health state using a vertical visual analogue scale, where 0 is “the worst imaginable” and 100 “the best imaginable” (EQ-VAS) [20]. The IES-R questionnaire evaluates three core symptoms of subjective distress, i.e., intrusion, avoidance and persistent hyperarousal, related to a stressful event (COVID-19 in our study population). Patients report how distressed or bothered they are by particular difficulties using a scale that ranges from 0 (“not at all”) to 4 (“extremely”). A total mean score ≥ 1.6 suggests symptom levels compatible with a diagnosis of post-traumatic stress disorder [21]. We also asked the patients to describe the most stressful aspect of their experience in ICU in a free text domain.

Data are reported as mean (SD), median [IQR], and frequency. They were compared with the Student’s *t*, the Mann–Whitney rank sum, or the Chi-square test, as appropriate (R version 3.6.1). The association between variables was studied with the Spearman’s correlation. A two-tailed p value < 0.05 was considered statistically significant.

## Results

**Fig. 1:**
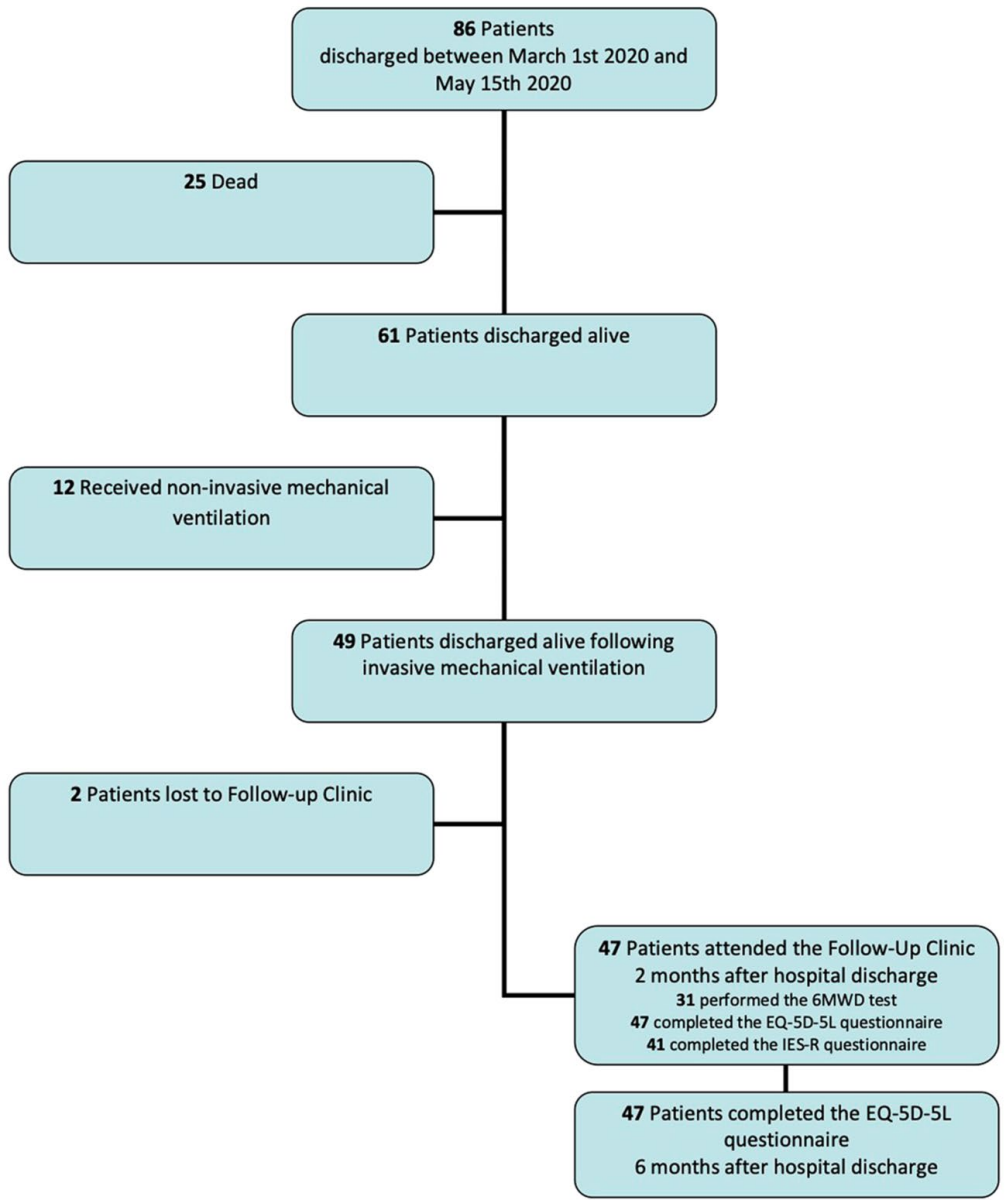
Enrollment of patients following hospital discharge. Between March 1st, 2020 and May 15th, 2020 a total of 86 laboratory confirmed COVID-19 ARDS patients were discharged from the hospital following an ICU stay. Of these 25 died during their ICU stay and 12 received noninvasive ventilation only. No deaths were observed after ICU discharge. A total of 49 patients were discharged alive following at least 48 h of mechanical ventilation in the study period. Overall, 31 (63%) patients performed the 6MWT, 47 (96%) completed the EQ-5D-5L questionnaire in person (33 patients) or by telephone (14 patients), and 41 (84%) completed the web-based IES-R questionnaire

From March 1st to May 15th, 2020, 49 patients treated with invasive mechanical ventilation in our ICU were discharged alive to home (28), rehabilitation (19), or a nursing facility (1). All patients initially discharged to rehabilitation were living at home or at temporary accommodations (due to isolation needs) at the time of follow-up. Two patients were lost to follow-up as no contact details were available. 47 patients are included in the study (Fig. 1). As shown in Table 1, these patients had a mean age of 59 ± 10 years and presented more commonly with a CFS score ≤ 3. Median early follow-up time was 49 [23–70] days from hospital discharge and 59 [41–86] days from ICU discharge. Four patients were discharged to a distant rehabilitation facility and were interviewed (EQ-5D-5L) by phone. 43 attended the hospital clinic. Of these, 31 patients performed the 6MWT and 12 did not; two as the walking course was not available as being used by other hospital services, while the patient attended our follow-up clinic (but completed the EQ-5D-5L in person), and 10 as no research team was available at the time. The latter were reached by phone to complete the EQ-5D-5L. Overall 31 (63%) patients performed the 6MWT and 47 (96%) completed the EQ-5D-5L questionnaire in person (33 patients) or by telephone (14 patients) and 41 (84%) filled-in the web-based IES-R questionnaire. As shown in the additional file, patients who did or did not attend our early follow-up program in person did not differ in their baseline characteristics. All 47 patients were alive at 6 months from hospital discharge, and completed a second iteration of the EQ-5D-5L.

**Table 1:** Baseline characteristics

	Study population (*n* = 47)
Demographic characteristics	
Age (years)	59 ± 10
Male n (%)	37 (79)
BMI at admission (kg/m^2^)	28.4 ± 5.3
APACHE II	6 [5–9]
SOFA	5 [3–6]
Charlson Comorbidity Index	2 [1–3]
Clinical Frailty Scale (CFS)	2 [2, 3]
Physiological variables at ICU admission	
PaO_2_/FiO_2_ admission	126 ± 43
paCO_2_ admission (mmHg)	46 ± 11
pH admission	7.40 ± 0.09
Treatments delivered in ICU	
NMBA (days)	4 [2–7]
Vasopressors n (%)	32 (65)
Prone n (%)	10 (21)
Corticosteroids n (%)	1 (2)
CRRT n (%)	3 (6)
Cumulative fluid balance (ml)	9 ± 322
Initial inpatient stay	
Hospital stay before ICU admission (days)	3 [1–5]
Invasive Mechanical Ventilation (days)	12 [6–17]
ICU length of stay (days)	15 [9–19]
Hospital length of stay (days)	29 [19–35]
Ventilator free days (day)	16 [11–22]

*BMI* Body Mass Index, *NMBA* neuro-muscolar blocking agent, *CRRT* continuous renal replacement theraphy, *ICU* intensive care unit

**Fig. 2:**
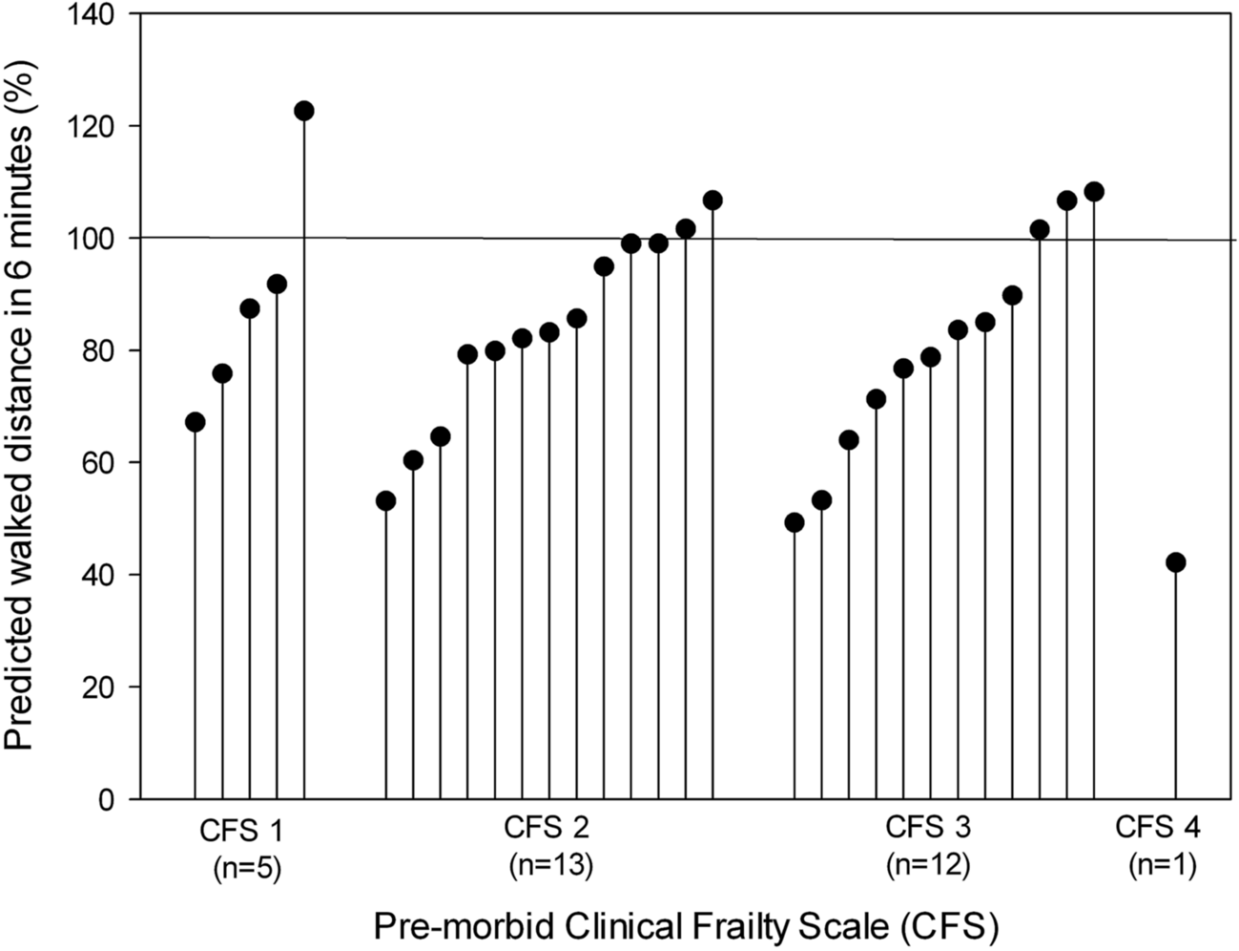
Individual data (black dots) are presented as percentage of predicted values for healthy subjects of same age, sex and body mass index. Patients are grouped according to their premorbid Clinical Frailty Scale (CSF)

The median distance walked in 6 min was 470 [406–516] m that is 83 [67–99]% of the predicted value (Fig. 1). Median difference between walked distance and the lower limit of normality (LLN) was 45 [− 38 to 121] m and 11/31 (35%) patients had 6MWD values below LLN. Among subjects with a premorbid CSF score of 1 or 2, the walked distance was 489 [417–553] m; 84 [74–99]% of the predicted value and 5/18 (28%) patients with premorbid CFS 1–2 had 6MWD values below LLN. In our case series there was no difference in this performance when explored by means of pre-existing clinical frailty score (p = 0.373) (Fig. 2). Of note, the median distance walked in 6 min (expressed as % of the predicted value) was greater in patients with longer time to follow up (rho = 0.415 p = 0.015). During the test, none of the patients had an arterial oxygen saturation < 88% and only 10 graded their dyspnea as moderate or worse (Borg scale > 2). Details about distance and physiological data measured before and after the test are presented in the supplemental digital content.

At the early follow-up one-third of patients presented with a moderate to severe impairment in at least one domain of their quality of life, with mobility and usual activities being the most frequent impairments and pain and depression/anxiety the most severe. None were on supplementary oxygen, wheelchair-bound, or bedridden at the time of follow-up. At 6-month follow-up one-third of patients still had a moderate to severe impairment in their quality of life. Patient mobility improved over time and everyone was able to self-care; however, usual activities and depression/anxiety were still the most affected and the most severe. Among patients with premorbid CFS 1–2, 6/26 (23%) presented moderate-severe impairment in their quality of life in at least one domain, 1 felt extremely severe pain at early follow-up; at 6 months 7/26 (28%) patients with premorbid CFS 1–2 had moderate-severe impairment in their quality of life in at least one domain, with only one patient having severe impairment in both the mobility and pain domains. Overall, the median early EQ-VAS was 80 [70–90] out of 100, and improved to 85 [77.5–90] at 6 months. Quality of life results from the early follow-up as well as the 6 months are presented in detail in Table 2.

**Table 2:** EQ-5D-5L for the study population. Frequencies and proportions are reported by dimension and level (*n* = 47)

	Mobility n (%)		Self-care n (%)		Usual activities n (%)		Pain/discomfort n (%)		Anxiety/depression n (%)	
	Early	6 months	Early	6 months	Early	6 months	Early	6 months	Early	6 months
Level 1 (no problems)	28 (60)	38 (81)	41 (87)	47 (100)	25 (53)	33 (70)	27 (57)	26 (55)	32 (68)	28 (60)
Level 2 (slight problems)	12 (26)	5 (11)	4 (9)	0	14 (30)	7 (15)	12 (26)	16 (34)	9 (19)	9 (19)
Level 3 (moderate problems)	7 (14)	2 (4)	1 (2)	0	7 (15)	5 (11)	5 (11)	4 (9)	4 (9)	9 (19)
Level 4 (severe problems)	0	2 (4)	1 (2)	0	0	0	2 (4)	1 (2)	2 (4)	1 (2)
Level 5 (extreme problems/unable to do)	0	0	0	0	1 (2)	2 (4)	1 (2)	0	0	0

On average, the IES-R total score was 1.94 ± 0.75; it was greater than the threshold for concern of 1.6 in 27 (66%) respondents. Mean sub-scores were 2.27 ± 0.84 for intrusion; 1.77 ± 0.77 for avoidance; and 1.73 ± 1.07 for hyperarousal. The three most common causes of extreme distress during the ICU stay were “being afraid of dying” (17 respondents), “being unable to communicate with loved ones” (15 respondents), “being worried for my loved ones” including fear of having infected them (11 respondents). Details about patient’s inclusion in the study and supplementary results are presented in the additional file.

## Discussion

This case-series describes follow-up data of a cohort of patients with severe, invasively mechanically ventilated COVID-19 patients early (circa 2 months) and 6 months following hospital discharge. Patients discharged alive from hospital presented with a reduced functional capacity at 2 months as described by a reduced distance on the six-minute walking test; however, no exercise-induced hypoxia was observed, and no patients had to interrupt the test; a mild-to-moderate reduction in quality of life was observed at 2 and 6 months with mobility, when dissecting the components of HRQoL self-care and usual activities showed the greatest improvement and depression/anxiety worsening over time. Moreover, a significant prevalence of PTSD symptoms were reported at 2 months from hospital discharge.

In our study population, the average distance walked in 6 min was 470 [406–516] m or 83 [67–99]% of its predicted value and exertional desaturation or dyspnea were very uncommon. Patients recovering from critical illness frequently perform much worse, with a mean distance walked in 6 min of 361 (95%-confidence intervals 321–401) m or 50–70% of the predicted value, 3 months after hospital discharge [22]. In a previously published cohort of severe ARDS patients [22], results were even worse (281 [55–454] m or 49%) possibly as a result of longer mechanical ventilation (21 days) and ICU days (25 days) resulting in more severe muscle wasting and weakness. In a recent case-series of severe COVID-19 who did not need invasive mechanical ventilation patients walked a median of 380 [180–470] meters at 2 months from hospital discharge, a similar time frame to our cohort, and did not present with exertional hypoxia or dyspnea. There, authors conclude that although with a degree of functional impairment, COVID-19 patients who did not require invasive mechanical ventilation were unlikely to develop long-term pulmonary impairments after hospital discharge, whereas fatigue was a common symptom [5]. Patients who received invasive or noninvasive (including high flow oxygen) respiratory support presented in a 6-month follow-up study from China [23] walked similar distances to our cohort with a median 479 [434–515.5] m.

Taken together, COVID-19 survivors of invasive mechanical ventilation, present mild functional impairment, similar or even better in our case, to some cases not-requiring mechanical ventilation. When compared to more traditional ARDS case-series, less pronounced physical impairment may be explained by the lower burden of premorbid disease and frailty, less acute and severe dysfunction of extra-pulmonary organ systems (lower APACHE and SOFA score at admission), and shorter length of ICU and hospital stay [24].

Regarding quality of life one-third of patients presented with a mild to moderate impairment of at least one domain in their quality of life. Compromised domains of quality of life changed over time. For instance, at the early follow-up up to 40% of patients reported mild to moderate impairment in the mobility domain and 45% with performing usual activities. At 6-month quality-of-life follow-up most patients reported no problems in the mobility domain, likely a result of long term rehabilitation and reintroduction of family life, no one reported issues with the ability of taking care for themselves, 35% were still limited in their ability to perform their usual activities. 40% reported issues with anxiety and depression with no improvement from the previous follow-up. This latter is in line with the early results of the IES-R questionnaire, and it might be influenced as well by the fact that the 6-month follow-up of this early cohort of patients falls during the “second wave” and new lockdown of our region.

Interestingly, the overall assessment of the patient’s health-related quality of life as expressed by EQ-VAS was in line with normative values for the general adult population of our region (EQ-VAS: 80 [70–90]) (15) and remained stable at 6 months [25]. This suggests that the perceived quality of life of the discharged population is similar to that of the general population of our region and, accordingly, it might have been the same also before patients’ hospital stay for COVID-19. A variety of HRQoL have been reported with varying results. In a cohort of 33 non-intubated COVID-19 patients, Daher et al. [5] found lower EQ-VAS scores (i.e., a median value of 63 [53–80]) at 2 months from hospital discharge. Moreover, a recent case-series of 78 COVID-19 patients centering its research on Patient Related Outcomes (PROM) found similar reductions in quality of life, particularly linked with pre-existing conditions as measured by Charlson comorbidity index, with no significant difference between ventilated and non-ventilated patients [26]. However, the largest 6-month follow-up study to date, a cohort of 1733 patients from China [23] showed similar results to our population. Considering only the sub-cohort of patients who received respiratory support (invasive or noninvasive *n* = 122) they present an EQ-VAS of 80 [70–87.5], with the most relevant compromised domains of HRQoL being pain (41%) and depression/anxiety (32%). Our results are more severe in the latter and we believe this is due to our cohort including exclusively patients receiving invasive mechanical support.

The overall reduction in quality of life seen in survivors of severe COVID-19 is likely multifactorial, due to the combination of prolonged hospital stay, pre-existing conditions and post-viral fatigue, a symptom very often reported by survivors [4] and already shown in previous SARS and MERS epidemics [27]. However, the perception of quality of life and the performance of quality of life measurement tools is subject to cultural and social factors and is probably best compared to local normative standards than to other case-series from different geographical areas.

Regarding post-traumatic stress symptoms, the prevalence and severity of intrusion, avoidance and hyperarousal symptoms linked to COVID-19 were extremely common, with two thirds of patients at risk for post-traumatic stress disorder, compared to approximately one-fifth in other series of critically ill patients [28, 29]. These values are much higher than expected and from those in COVID-19 patients outside the ICU. It is likely that the ICU environment, the invasiveness of treatment and the isolation might have played a role in this. As suggested by patients themselves, this may have been related to the additional burden posed by the pandemic, due to the compulsory isolation and significant worry about loved ones. When interpreting these data it is important, however, to note that, assessment of PTSD symptoms was performed during a health crisis, and it’s a single point in time. Long term follow-up, especially after the crisis has ceased, will be needed to understand if the symptoms persist and the true prevalence of this problem.

Overall, our patients suffering from acute hypoxemic respiratory failure from COVID-19, presented an acceptable cardiopulmonary performance, better than in patients recovering from other forms of ARDS, and mild forms of physical impairment, mildly reduced overall quality of life, and a high proportion of PTSD symptoms at time of assessment. Even if the impact of severe COVID-19 on functional capacity and quality of life can seem less severe than those of other critical illnesses, they may still have an important social and economic impact. The number of patients admitted to the ICU with COVID-19 is, and probably will continue to be, very large. Many patients might suffer from persisting physical and psychological disability, including those with good premorbid health and functional status. In our case series 28% of patients with good premorbid functional status (CFS 1–2) could not reach their expected lower limit of normality distance at 6-min walking and presented with impaired domains in their perception of health-related quality of life. At a population level, considering the global prevalence of the disease, these findings may predict that a large number of subjects will experience some post-discharge physical and/or psychological impairment, with substantial physical, psychological, social and economical burden on the individuals themselves, their caregivers and society. At both early and late follow-up, patients recovering from COVID-19 in our cohort present decrements in quality of life. There is some improvement between 2 and 6 months, but it is not complete. This means, we believe, that (a) there is room for rehabilitation interventions to improve recovery; and (b) that evaluations of such interventions should consider the likelihood that EQ-5D scores will change over time even in the absence of an intervention, so randomization or other control for changes over time are essential to an unbiased evaluation of any intervention.

The limitations of this study deserve comment. First, our study population was small, quite young and previously fit. Our results should then be considered preliminary and may not be valid for other groups of patients with poorer baseline health status with severe COVID-19. In addition, the initial organ severity (as represented by SOFA score) was lower than other COVID-19 literature, suggesting most of our patients might have experienced single-organ disease, and this might explain our relatively high ventilator free days as compared to other works such as Tomazini et al. [30].

Second, we could not achieve an exact 2-month follow-up for all the patients, our population presents a median 2-month follow-up, with appointments being given between 1 and 2 and half months from hospital discharge. This was due to the need to accommodate patients' logistic requests in scheduling follow-up visits, considering many had long travel distances and travel restrictions were in place, and the ability of the study investigator to run the clinic while not busy in other clinical duties. The clinic is run pro-bono on physician’s own time.

Third we could not compare our results with patient baseline, pre-existing quality of life values as these were not available/not collected. Fourth, we could not perform a repeated 6MWT due to the new lockdown affecting our country. Fifth, due to the anonymity of the IES-R we could not correlate the results of this test with other domains of quality of life. Finally, a longer follow-up time is required to understand whether functional and psychological impairment is persistent or not.

## Conclusions

In conclusion, patients recovering from severe COVID-19 requiring invasive mechanical ventilation surviving hospital discharge present with early mild to moderate functional impairment, mildly reduced quality of life from hospital discharge with an overall improvement of mobility, self-care and the ability of performing usual activities, while a worsening of pain and depression/anxiety symptoms at 6 months and a large proportion of symptoms of post-traumatic distress soon after hospital discharge. Our findings reflect the characteristics of our population, hence our results must be taken cautiously. Further research is required to understand the magnitude of the impact of COVID-19 across different health statuses and health-care systems worldwide to confirm our research data.

## Data Availability

All data generated or analysed during this study are included in this published article [and its supplementary information files].
